# Associations between immigration background, adverse childhood experiences, and depressive symptoms in adulthood in immigrants and descendants of immigrants in France: a mediation analysis

**DOI:** 10.1186/s12991-025-00612-7

**Published:** 2026-02-17

**Authors:** Kasra Zarei, Pamela J. Surkan, Judith van der Waerden, Emmanuel Wiernik, Siddartha Aradhya, Anna-Clara Hollander, Kelvin Choi, Maria Melchior

**Affiliations:** 1 Division of Intramural Research, National Institute on Minority Health and Health Disparities, Bethesda, 20892-5465 MD USA; 2 Sorbonne Université, L’Institut national de la santé et de la recherche médicale (INSERM), Institut Pierre Louis d’Epidémiologie et de Santé Publique (IPLESP), Epidémiologie Sociale, Santé Mentale, Addictions (ESSMA), Cedex 12, Paris, 75571 France; 3 Department of Global Public Health, Karolinska Institutet, 17176 Stockholm, Sweden; 4 Department of International Health, Social and Behavioral Interventions Program, Johns Hopkins Bloomberg School of Public Health, Baltimore, MD 21205 USA; 5 Université Paris Cité, Paris Saclay University, Université de Versailles Saint-Quentin-en-Yvelines, INSERM UMS 011 « Population-based Cohorts Unit », 91190 Paris, France; 6 Department of Sociology, Stockholm University Demography Unit, 10691 Stockholm, Sweden

**Keywords:** Geographic area of origin, Depression, Adverse childhood experiences, Mediation

## Abstract

**Introduction:**

In France, 10% of the population are immigrants and another 11% are children of immigrants. Both have worse mental health than the native French. The role of adverse childhood experiences (ACE) in immigrants’ mental health is not well characterized. We aimed to examine associations between immigration background, ACEs, and depressive symptoms.

**Methods:**

Data came from the baseline and 2020 follow-up questionnaires of the French CONSTANCES study (*n* = 116,495), a national cohort. The exposure was immigration background categorized by immigration generation (1st : immigrants; 2nd : French-born with ≥ 1 immigrant parent; and native French) and the geographic origin of the participant (1st generation) or ≥ 1 parent (2nd generation). The mediator was experiencing ACEs. The outcome was depressive symptoms ascertained with the Center for Epidemiologic Studies Depression scale at study inclusion. Mediation analysis using multivariable logistic regression and path analysis (PA) was used to assess associations between the exposure, mediator, and outcome, overall and stratified by sex, minimally adjusting for age and sex or adjusting for all covariates.

**Results:**

The prevalence of depressive symptoms was 18.5%. In minimally adjusted models, compared to native French, there were higher odds of depressive symptoms in 1st and 2nd generation adults except those with ≥ 1 parent from Asia. Mediation effects of ACEs from PA ranged from 0.03 to 0.10. In the fully adjusted model including after adjusting for experiencing ACEs, only immigrants from North Africa had significantly increased odds of depressive symptoms (AOR = 1.52, 95%CI: 1.29, 1.79).

**Conclusions:**

In France, non-native adults have higher prevalence and odds of depressive symptoms than the native French, with ACEs having a significant mediating effect.

**Supplementary Information:**

The online version contains supplementary material available at 10.1186/s12991-025-00612-7.

## Key messages

- Compared to the native French, there were generally higher odds of depressive symptoms and experiencing ACEs in immigrants and descendants of immigrants.
- Experiencing ACEs may have a mediating effect on depressive symptoms among most groups of immigrants and descendants of immigrants.
- Our findings from a large, diverse cohort have implications for risk factors to prioritize to advance psychosocial health and research among immigrants and their descendants, in relation to both origin-specific and general factors related to depression and ACEs.

**Supplementary Information:**

The online version contains supplementary material available at 10.1186/s12991-025-00612-7.

## Background

Migration is a longstanding human phenomenon, and in some countries, immigrants and their descendants represent a significant portion of the population. In France, immigrants make up around 10% of the population [1–3], and approximately 11% of the population are 2nd -generation individuals (i.e., France-born individuals with ≥ 1 immigrant parent) [4]. Mental health can vary across immigration status [5–9], and in France, immigrants can have worse mental health than the native French [10, 11] including mood disorders such as depression [12].

Adverse childhood experiences (ACEs) refer to potentially traumatic experiences that a child may experience [13], and could potentially contribute to the increased prevalence of mental illness and depression in immigrants and descendants in France. There is substantial evidence demonstrating associations between ACEs and poor health outcomes [13–15] across the lifespan in different countries of all income levels (high to low and middle income) [16]. Globally, cumulative and individual ACEs such as violence and abuse have significant impacts on health outcomes including smoking, substance use and mental ill health [17–19]. Some data suggests that population estimates of individual ACEs can vary by country or the country income category [16]. For example, the prevalence of ACEs like violence is high in some low-income countries [20]. It is possible that individuals from middle to lower income countries are more likely to experience certain ACEs, including abuse and neglect compared to people from high income countries, but this relationship has not been clearly established. Some prevalence estimates of experiencing cumulative ACEs have appeared relatively stable across countries that have been included in global surveys [16]. As ACEs can vary by country or social and structural factors [21], it is possible that ACEs can be differentially experienced by immigrants and subsequently transmitted across generations [22]. Beyond ACEs, other socioeconomic factors can contribute to adverse mental health outcomes among immigrants and their descendants including income, labor market factors, and education [23–25].

In France, there is a significant prevalence of ACEs [18, 26–28], but little information on ACEs by immigration background, and the relationship to subsequent mental health including depression. Studies in the U.S., for example, have found varying burdens of ACEs in immigrants, children of immigrants, and the general population, with some 1st and 2nd generation groups of different ancestries having increased prevalence of ACEs [29]. In France, across three immigration generations, immigrants and their descendants have a greater prevalence of having any mood disorder including depression [12], but specific information such as geographic origin by immigration generation has not always been studied. Understanding underlying factors that influence depression in immigrants and their descendants in France, including ACEs, is important for promoting immigrant health.

The aim of this study is to examine associations between individuals’ immigration background (geographic origin and immigration generation), ACEs, and depressive symptoms in adulthood and whether there is a mediating effect of ACEs. Secondary aims include examining these associations by sex, as depression and experience of certain ACEs including sexual and physical abuse [30] can vary by gender [31, 32], and assessing the role of sociodemographic factors as covariates. The current study will examine these associations using up-to-date, national data from the French CONSTANCES cohort [33]. We hypothesize that there are significant differences in the prevalence of ACEs and depressive symptoms, with immigrants and their descendants having higher prevalence of both compared to the native population. This hypothesis is partially based on factors including differences in exposures to a different environment in childhood including household dysfunction and challenges [16], limited economic opportunities [4], and experiences of discrimination and difficulties integrating into society [34]. We also subsequently hypothesize that ACEs mediate the relationship between immigration background and depressive symptoms, in a way that is comparable across both sexes.

## Methods

### Data sources

Data were drawn from participants who completed the 2020 follow-up questionnaire (*n* = 116,495) of the French CONSTANCES study. CONSTANCES is a general-purpose cohort for epidemiological research that began recruitment in 2012 on a rolling basis afterwards [33, 35, 36], designed as a randomly selected sample of the general French adult population, aged 18–69 years, that is designed to be representative in terms of age, gender, and socio-economic status of the target population, but may differ due to selection bias at study inclusion [33]. However, the CONSTANCES study is not weighted to be nationally representative and does not have nationally representative complex survey weights available to use for this analysis.

### Exposure

The exposure was defined as the participant’s self-reported immigration background based on several variables: the geographic area of origin of the participant and their parents, and the participant’s nationality. Response categories for geographic origin included: “Metropolitan France”, “French Overseas Territories/Departments” [DOM-TOM], “Europe”, “North Africa” [NA], “Sub-Saharan Africa” [SSA], “Asia”, “Other”, and “Don’t want to answer” based on the most frequent areas of origin of immigrants in France.

Immigration generation was defined, consistent with the relevant literature, based on the participant and parental area of birth: 1 st generation = immigrants (both the participant and ≥ 1 parent were born outside of mainland France), 2nd generation = participants born in mainland France with ≥ 1 immigrant parent, and native French = both participants and their parents were born in France, or the participant was born outside of France, but both parents were born in France. While there are differences in households with individuals born in France with one vs. two immigrant parents, there are some hypothesized closer similarities between them regarding shared origin and related factors (sociocultural factors and identities, etc.) compared to individuals from France whose parents were both born in France. For 1st and 2nd generation participants, further categories were defined based on the geographic origin of the participant (1st generation) or ≥ 1 parent (2nd generation). We used data on nationality (responses: “French by birth”, “Naturalised French”, and “Foreign national”) to categorize 1st generation participants who were born outside of France (mainland or overseas territories), but who were “French by birth” in the reference group (native French). In total, there are 13 categories to represent the participant’s immigration background.

### Outcome

The outcome was depressive symptoms at the time/year of inclusion in the CONSTANCES study assessed with a score of ≥ 16 on the original version of the Center for Epidemiologic Studies Depression Scale (range: 0–60) [37]. The cutoff score of 16 on the CES-D has previously demonstrated optimized sensitivity and specificity [38], although studies specific to France have shown gender-specific cutoffs (i.e. higher cutoffs in females) may further optimize sensitivity and specificity in French samples [39]. We dichotomized depressive symptoms as an outcome to use for statistical modeling, using a consistent cut-off score given the presence of varying immigration backgrounds that may have not necessarily been validated using gender-specific cut-off scores. The cut-off score of 16 was also used to better compare the findings with the international literature.

### Mediator

Information on ACEs before the age of 18 years was collected during the 2020 follow-up questionnaire of CONSTANCES. ACEs used in this study are included in Table 1 [13]. Responses were used to determine if participants experienced any ACEs (0 vs. 1+), as certain types of ACEs are interrelated, and determine a collapsed ACE count (0, 1, 2, 3, 4+), in accordance with previous ACE research [13, 29]. These aggregate ACE measures were used as the mediator.

**Table 1:** Adverse childhood experiences measured among participants who completed the 2020 follow-up questionnaire of the CONSTANCES study (France, 2012-present)

Prior to age 18 years, experienced:
• Loss of a parent – any parental separation, divorce, or death
• Family having regular financial difficulties.
• Living with someone who suffered from depression, mental illness, or was suicidal.
• Living with someone who had excessive alcohol consumption or alcohol dependence or was using illicit drugs or who was taking excessive amounts of medication (outside of a medical setting).
• Living with someone who has been sentenced to prison (or other type of correctional facility) with or without a sentence arrangement.
• Witnessing or experiencing household physical violence done by parents or other adults at the home in the form of:
• Witnessing physical violence: parents or other adults at home slap, punch or kick each other at least once.
• Experiencing physical violence: a parent or other adult in the household hit, beat, kick or physically hurt the participant at least once.
• Experiencing verbal/emotional abuse: a parent or other adult in the household yelled, insulted, or belittled the participant at least once.
• Experiencing sexual abuse consisting of:
• Someone made the participant suffer sexual touching.
• Someone tried to obtain sexual favors from the participant at least once.
• Someone forced the participant to have sex at least once.

Responses were classified as “Yes; at least once or one” or “Never/No”, with responses of “I don’t know, I’m not sure”, or “I don’t want to answer” treated as missing. The number of responses of “Yes; at least once or one” were added to determine if children experienced any ACEs (0 vs. 1+) and determine an ACE count (0, 1, 2, 3, and 4+).

### Covariates

From sociodemographic information collected in the CONSTANCES inclusion questionnaire, covariates were identified a priori based on those with demonstrated associations (*p* < 0.05) with immigration background and depressive symptoms and included educational level (highest diploma received), income (household’s average net monthly income), marital status (married or in a civil partnership vs. not), employment (“has a job, including on leave” or “retired or no longer in business” vs. other), and current occupational grade or occupational grade held for the longest time if not currently working (≥ intermediate professional or business owner vs. not). Covariates in this study were dichotomized due to the software and statistical modeling used. Generally, information with greater granularity was limited for the purposes of creating dichotomized covariates. For a separate analysis involving 1st generation participants, time lived in France was dichotomized as < 10 vs. ≥10 years.

### Statistical analyses

We estimated the distributions of the study exposure, mediator, outcome, and covariates, and the prevalence and mean estimates of the mediator, outcome, and covariates by immigration background. The Baron and Kenney approach [40] was first used to assess associations between immigration background, ACEs, and depressive symptoms, adjusting for sociodemographic factors (age, sex, education, income, marital status, employment situation, and occupational grade). The Baron and Kenny method was used as a preliminary analysis to evaluate the appropriateness for performing a causal mediation analysis. Specifically, we implemented the following multivariable logistic regression models with the native French serving as the reference group:

1. Association between immigration background and depressive symptoms, adjusting for age and sex.
2. Association between immigration background and experiencing ACEs, adjusting for age and sex.
3. Association between experiencing ACEs and depressive symptoms, adjusting for age and sex.
4. Association between immigration background and depressive symptoms, adjusting for experiencing ACEs, age, and sex.
5. Repeat all models adjusting for all covariates and with the ACE count in steps 3 and 4.

**Fig. 1:**
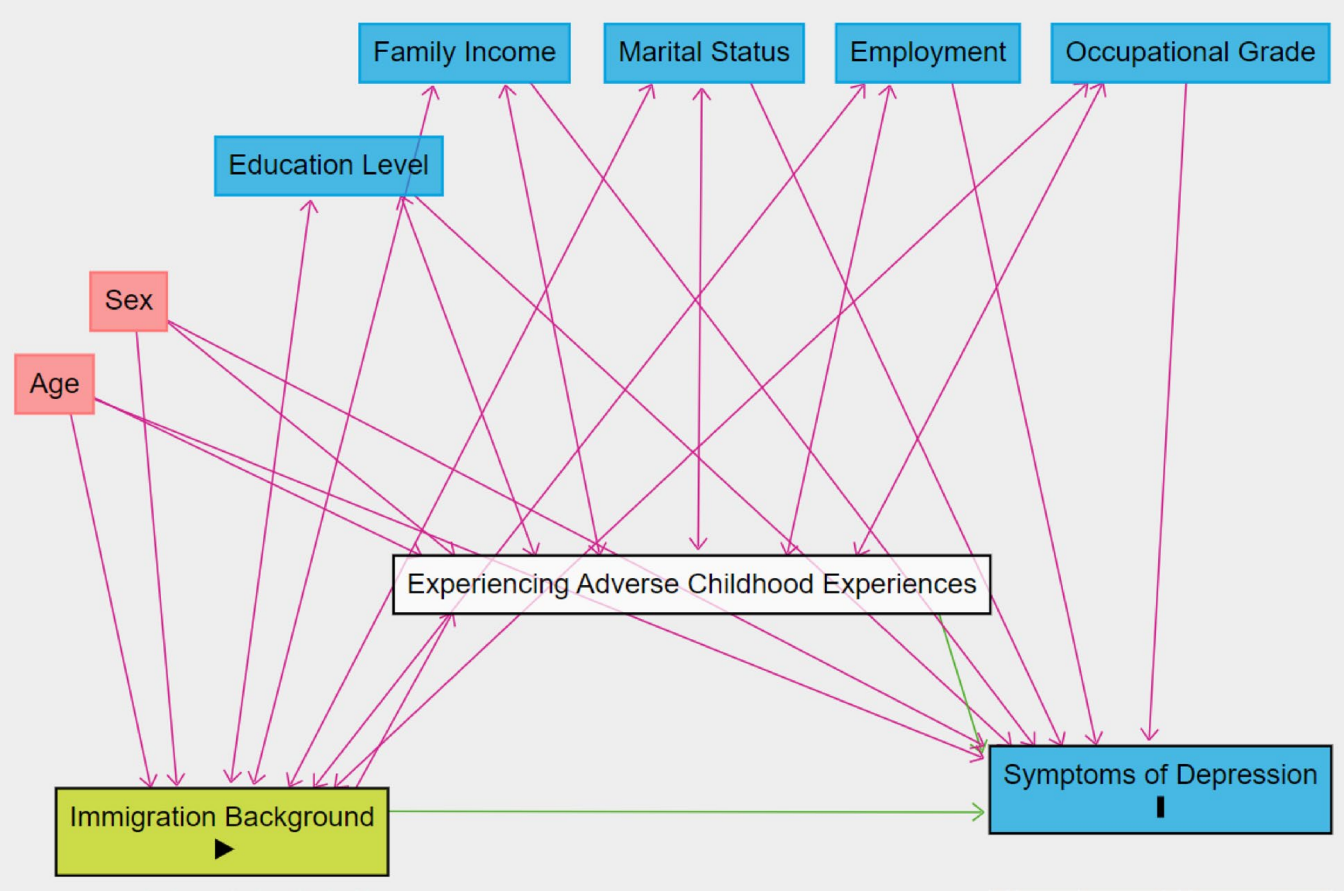
Illustration of the conceptual model between the exposure (immigration background), mediator (experiencing adverse childhood experiences), outcome (depressive symptoms in adulthood), and covariates

We ran both a minimally adjusted (age and sex) and fully adjusted (all covariates) model as some demographics in the fully adjusted model may also partially function as mediators, although assessing these relationships was not the objective of the study. Mediation analysis was also performed by applying a path analysis (PA) to estimate mediation effects [41]. Figure. 1 illustrates the study’s conceptual model [42]. It is possible that covariates including education, income, marital status, employment situation, and occupational grade influence immigration background (for instance, more educated, higher income, employed, non-single individuals are more likely to be immigrants or children of immigrants). In a separate analysis involving 1 st generation participants only, we evaluated the association between immigration background, time lived in France and the outcome.

Statistical analyses were conducted using available data only and using imputed data in SAS^®^ software version 9.4 (SAS Institute: Cary, NC). Mediation analyses with PA were performed using MPlus software, and mediation effects were estimated using the MODEL INDIRECT statement [43] with a statistical significance level of 0.05.

## Results

Table 2 shows the distributions of study variables. Most participants (83.9%) were native French, followed by 2nd generation of European origin (5.6%), and 2nd generation of NA origin (2.5%). The prevalence of depressive symptoms was 18.5%, and the average ACE count was 1.5 (standard deviation = 1.5). About 65.0% of participants reported experiencing ACEs. Supplemental Table 1 reports the prevalence of study variables by immigration background.

**Table 2:** Distributions of demographics and study variables among participants (*n* = 116,495) who completed the 2020 follow-up questionnaire of the CONSTANCES study (France, 2012-present). The initial descriptive statistics presented here are based on the original, non-imputed data only

Variable	Median (IQR)	Unweighted *N* (%)
**Information collected at study inclusion**		
**Exposure**		
Immigration background		
Native French, 3rd + generation		97,760 (83.9%)
Overseas territories, 1 st generation		639 (0.5%)
Europe, 1 st generation		2697 (2.3%)
North Africa, 1 st generation		827 (0.7%)
Sub-Saharan Africa, 1 st generation		489 (0.4%)
Asia, 1 st generation		472 (0.4%)
Other, 1 st generation		727 (0.6%)
Overseas Territories, 2nd generation		483 (0.4%)
Europe, 2nd generation		6478 (5.6%)
North Africa, 2nd generation		2929 (2.5%)
Sub-Saharan Africa, 2nd generation		211 (0.2%)
Asia, 2nd generation		458 (0.4%)
Other, 2nd generation		592 (0.5%)
Missing		1733 (1.5%)
**Covariates**		
Age at study inclusion (years)	49.5 (21.5)	-----
Sex		
Male		52,592 (45.1%)
Female		63,903 (54.9%)
Family average net monthly income		
<2100 euros		19,529 (16.8%)
≥2100 euros		89,616 (76.9%)
Missing		7350 (6.3%)
Highest level of education completed		
French baccalaureate (secondary school) or lower		41,573 (35.7%)
Higher than French baccalaureate (secondary school)		73,179 (62.8%)
Missing		1743 (1.5%)
Marital status		
Not married (never married, separated, divorced, widow.er)		41,626 (35.7%)
Married or in a civil partnership		72,925 (62.6%)
Missing		1944 (1.7%)
Employment		
Has a job or retired or no longer in business		103,463 (88.8%)
Other employment situation		10,404 (8.9%)
Missing		2628 (2.3%)
Occupational grade		
≥ Intermediate professional or craftsperson, shopkeeper, or business owner		74,172 (63.7%)
< Intermediate professional or craftsperson, shopkeeper, or business owner		36,540 (31.4%)
Missing		5783 (5.0%)
**Outcome**		
Depression symptoms (Center for Epidemiologic Studies Depression Scale ≥ 16)		
No		89,689 (77.0%)
Yes		21,562 (18.5%)
Missing		5244 (4.5%)
**Information collected during study follow-up**		
**Adverse childhood experiences (ACEs)**		
ACE count	1.0 (2.0)	-----
Any ACE experienced		
No		39,587 (34.0%)
Yes		75,743 (65.0%)
Missing		1165 (1.0%)
ACE count (collapsed)		
0		39,587 (34.0%)
1		27,502 (23.6%)
2		21,050 (18.1%)
3		13,641 (11.7%)
4+		13,550 (11.6%)
Missing		1165 (1.0%)
**Specific ACEs**		
Parental separation or death		
No		92,527 (79.4%)
Yes		20,205 (17.3%)
Missing (includes “don’t know/not sure” or “not involved”)		3763 (3.2%)
Family financial difficulties		
No		80,694 (69.3%)
Yes		19,820 (17.0%)
Missing (includes “don’t know/not sure”)		15,981 (13.7%)
Household member with mental illness		
Never		86,729 (74.4%)
Yes		20,780 (17.8%)
Missing (includes “don’t know/not sure” or “don’t want to answer”)		8986 (7.7%)
Household member with alcohol or drug abuse		
Never		93,629 (80.1%)
Yes		21,218 (18.2%)
Missing (includes “don’t know/not sure” or “don’t want to answer”)		2008 (1.7%)
Household member incarcerated		
Never		112,414 (96.5%)
Yes		1296 (1.1%)
Missing (includes “don’t know/not sure” or “don’t want to answer”)		2785 (2.4%)
Verbal abuse from household member		
Never		62,354 (53.5%)
Yes		42,796 (36.7%)
Missing (includes “don’t know/not sure” or “don’t want to answer”)		11,345 (9.7%)
Sexual abuse from household member		
Never		97,021 (83.3%)
Yes		13,605 (11.7%)
Missing (includes “don’t know/not sure” or “don’t want to answer”)		5869 (5.0%)
Witnessing or experiencing household physical violence		
Never		81,028 (69.6%)
Yes		33,475 (28.7%)
Missing (includes “don’t know/not sure” or “don’t want to answer”)		1992 (1.7%)

### Mediation analysis steps

#### Association between immigration background and depressive symptoms, adjusting for age and sex

In the minimally adjusted models (Table 3), compared to the native French, there were higher odds of depressive symptoms for generally all groups of participants except 2nd generation participants from Asia (AOR range: 1.11–1.97, Table 3).

**Table 3:** Associations between immigration background, adverse childhood experiences, and depressive symptoms in adulthood in the CONSTANCES study (logistic regression analyses adjusted for age and sex (*), or for age, sex, education, family average net monthly income, marital status, employment situation, and occupational grade (**); adjusted odds ratio [AORs], 95% confidence intervals [95% CI])

Immigration Background/Model	Associations between immigration background, experiencing any ACE and demographics – AOR (95% CI)*	Associations between immigration background, depressive symptoms and demographics – AOR (95% CI)*	Associations between immigration background, depressive symptoms, experiencing any ACE and demographics – AOR (95% CI)*	Associations between immigration background, depressive symptoms, ACE count (0–4+) anddemographics – AOR (95% CI)*	Associations between immigration background, experiencing any ACE and demographics – AOR (95% CI)**	Associations between immigration background, depressive symptoms and demographics – AOR (95% CI)**	Associations between immigration background, depressive symptoms, experiencing any ACE and demographics (95% CI)**	Associations between immigration background, depressive symptoms, ACE count (0–4+) anddemographics (95% CI)**
**Reference: native French (3rd + generation)**								
**Overall**								
Overseas territories, 1 st generation	**2.21 (1.77–2.77)**	**1.62 (1.35–1.94)**	**1.49 (1.24–1.79)**	**1.37 (1.14–1.65)**	**2.12 (1.69–2.65)**	**1.31 (1.09–1.58)**	**1.22 (1.01–1.47)**	1.14 (0.94–1.38)
Europe, 1 st generation	**1.34 (1.23–1.47)**	**1.11 (1.01–1.22)**	1.07 (0.97–1.17)	1.01 (0.92–1.12)	**1.33 (1.22–1.45)**	1.05 (0.95–1.16)	1.01 (0.92–1.12)	0.96 (0.87–1.06)
North Africa, 1 st generation	**1.42 (1.21–1.67)**	**1.97 (1.68–2.31)**	**1.89 (1.62–2.23)**	**1.86 (1.58–2.19)**	**1.36 (1.15–1.59)**	**1.59 (1.35–1.87)**	**1.54 (1.31–1.81)**	**1.52 (1.29–1.79)**
Sub-Saharan Africa, 1 st generation	**1.93 (1.50–2.49)**	**1.68 (1.36–2.08)**	**1.56 (1.26–1.94)**	**1.47 (1.18–1.82)**	**1.81 (1.41–2.33)**	1.20 (0.96–1.50)	1.13 (0.91–1.41)	1.08 (0.86–1.36)
Asia, 1 st generation	1.19 (0.97–1.46)	**1.34 (1.08–1.66)**	**1.32 (1.07–1.64)**	**1.34 (1.08–1.67)**	1.16 (0.94–1.42)	1.18 (0.95–1.47)	1.17 (0.94–1.46)	1.19 (0.95–1.49)
Other, 1 st generation	**1.84 (1.52–2.23)**	**1.26 (1.05–1.50)**	1.18 (0.98–1.40)	1.08 (0.90–1.29)	**1.80 (1.49–2.19)**	1.16 (0.97–1.39)	1.09 (0.91–1.31)	1.01 (0.84–1.22)
Overseas territories, 2nd generation	**1.79 (1.42–2.25)**	**1.38 (1.10–1.73)**	**1.29 (1.03–1.62)**	1.19 (0.95–1.50)	**1.74 (1.38–2.18)**	1.21 (0.96–1.53)	1.14 (0.91–1.44)	1.06 (0.84–1.34)
Europe, 2nd generation	**1.25 (1.18–1.32)**	**1.13 (1.06–1.21)**	**1.10 (1.03–1.17)**	**1.07 (1.00–1.14)**	**1.24 (1.17–1.31)**	**1.10 (1.03–1.17)**	**1.07 (1.00–1.14)**	1.04 (0.98–1.11)
North Africa, 2nd generation	**1.45 (1.33–1.59)**	**1.16 (1.06–1.27)**	**1.11 (1.01–1.22)**	1.05 (0.95–1.15)	**1.44 (1.31–1.58)**	**1.12 (1.02–1.24)**	1.08 (0.98–1.18)	1.02 (0.93–1.13)
Sub-Saharan Africa, 2nd generation	**2.01 (1.39–2.91)**	**1.61 (1.18–2.18)**	**1.49 (1.10–2.03)**	**1.37 (1.01–1.88)**	**1.94 (1.34–2.82)**	**1.40 (1.03–1.92)**	1.31 (0.96–1.80)	1.22 (0.89–1.68)
Asia, 2nd generation	**1.39 (1.12–1.73)**	1.15 (0.91–1.45)	1.10 (0.87–1.39)	1.05 (0.83–1.33)	**1.36 (1.10–1.69)**	1.07 (0.84–1.36)	1.03 (0.81–1.31)	0.99 (0.77–1.26)
Other, 2nd generation	**1.69 (1.38–2.06)**	**1.30 (1.07–1.59)**	**1.23 (1.01–1.50)**	1.17 (0.96–1.43)	**1.63 (1.34–2.00)**	1.15 (0.94–1.40)	1.09 (0.89–1.33)	1.04 (0.85–1.28)
**Stratification by Sex**								
Female								
Overseas territories, 1 st generation	**2.50 (1.87–3.34)**	**1.54 (1.22–1.94)**	**1.41 (1.12–1.78)**	**1.29 (1.02–1.63)**	**2.38 (1.77–3.18)**	1.23 (0.97–1.56)	1.14 (0.89–1.44)	1.05 (0.82–1.34)
Europe, 1 st generation	**1.41 (1.25–1.59)**	1.07 (0.95–1.21)	1.03 (0.91–1.16)	0.98 (0.86–1.10)	**1.40 (1.24–1.58)**	1.03 (0.91–1.16)	0.99 (0.87–1.12)	0.94 (0.83–1.07)
North Africa, 1 st generation	**1.59 (1.21–2.10)**	**1.93 (1.54–2.42)**	**1.84 (1.47–2.32)**	**1.81 (1.44–2.28)**	**1.53 (1.16–2.01)**	**1.59 (1.26–2.00)**	**1.52 (1.21–1.93)**	**1.51 (1.19–1.91)**
Sub-Saharan Africa, 1 st generation	**1.94 (1.35–2.79)**	**1.64 (1.21–2.22)**	**1.53 (1.13–2.08)**	**1.45 (1.07–1.98)**	**1.83 (1.27–2.64)**	1.17 (0.86–1.60)	1.11 (0.81–1.51)	1.06 (0.77–1.46)
Asia, 1 st generation	0.99 (0.76–1.28)	1.12 (0.85–1.48)	1.13 (0.86–1.49)	1.15 (0.87–1.53)	0.98 (0.76–1.27)	1.01 (0.76–1.34)	1.01 (0.76–1.34)	1.04 (0.78–1.38)
Other, 1 st generation	**1.73 (1.36–2.19)**	1.16 (0.93–1.44)	1.09 (0.88–1.35)	1.01 (0.81–1.25)	**1.71 (1.35–2.17)**	1.08 (0.86–1.35)	1.02 (0.82–1.28)	0.95 (0.76–1.19)
Overseas territories, 2nd generation	**2.05 (1.48–2.83)**	**1.39 (1.07–1.81)**	1.30 (0.99–1.69)	1.19 (0.91–1.55)	**1.98 (1.43–2.74)**	1.22 (0.93–1.59)	1.14 (0.87–1.48)	1.05 (0.80–1.38)
Europe, 2nd generation	**1.33 (1.23–1.44)**	**1.13 (1.04–1.22)**	**1.09 (1.01–1.18)**	1.05 (0.97–1.14)	**1.32 (1.22–1.43)**	**1.10 (1.01–1.19)**	1.06 (0.98–1.15)	1.03 (0.95–1.12)
North Africa, 2nd generation	**1.44 (1.27–1.63)**	1.10 (0.98–1.24)	1.06 (0.94–1.19)	1.00 (0.89–1.12)	**1.42 (1.26–1.61)**	1.08 (0.96–1.22)	1.04 (0.92–1.17)	0.99 (0.87–1.11)
Sub-Saharan Africa, 2nd generation	**2.31 (1.37–3.89)**	**1.51 (1.02–2.23)**	1.39 (0.94–2.07)	1.27 (0.85–1.89)	**2.24 (1.33–3.77)**	1.37 (0.92–2.05)	1.27 (0.85–1.90)	1.17 (0.78–1.76)
Asia, 2nd generation	1.33 (0.99–1.78)	1.18 (0.90–1.55)	1.15 (0.87–1.51)	1.09 (0.83–1.44)	1.28 (0.95–1.73)	1.10 (0.83–1.45)	1.07 (0.81–1.41)	1.02 (0.77–1.36)
Other, 2nd generation	**1.69 (1.30–2.20)**	**1.30 (1.03–1.66)**	1.23 (0.97–1.57)	1.16 (0.91–1.49)	**1.64 (1.26–2.13)**	1.17 (0.92–1.50)	1.12 (0.87–1.43)	1.06 (0.83–1.36)
Male								
Overseas territories, 1 st generation	**1.94 (1.40–2.68)**	**1.77 (1.32–2.39)**	**1.64 (1.22–2.21)**	**1.53 (1.13–2.07)**	**1.86 (1.35–2.58)**	**1.51 (1.11–2.04)**	1.41 (1.03–1.92)	1.33 (0.97–1.82)
Europe, 1 st generation	**1.27 (1.12–1.44)**	**1.19 (1.01–1.41)**	1.15 (0.97–1.36)	1.09 (0.92–1.29)	**1.25 (1.10–1.42)**	1.10 (0.93–1.30)	1.07 (0.90–1.27)	1.02 (0.85–1.21)
North Africa, 1 st generation	**1.32 (1.08–1.62)**	**2.01 (1.62–2.49)**	**1.95 (1.57–2.42)**	**1.90 (1.53–2.37)**	**1.24 (1.01–1.53)**	**1.60 (1.28–2.00)**	**1.57 (1.25–1.96)**	**1.55 (1.23–1.94)**
Sub-Saharan Africa, 1 st generation	**1.93 (1.40–2.66)**	**1.72 (1.27–2.32)**	**1.59 (1.17–2.14)**	**1.47 (1.09–2.00)**	**1.77 (1.29–2.43)**	1.22 (0.89–1.67)	1.15 (0.84–1.57)	1.09 (0.79–1.50)
Asia, 1 st generation	**1.57 (1.11–2.23)**	**1.87 (1.31–2.67)**	**1.77 (1.23–2.53)**	**1.76 (1.23–2.53)**	**1.50 (1.06–2.13)**	**1.60 (1.11–2.30)**	**1.53 (1.06–2.21)**	**1.54 (1.06–2.23)**
Other, 1 st generation	**2.04 (1.51–2.76)**	**1.49 (1.10–2.02)**	**1.37 (1.01–1.85)**	1.22 (0.90–1.66)	**1.99 (1.47–2.70)**	1.35 (0.99–1.85)	1.26 (0.92–1.72)	1.14 (0.83–1.56)
Overseas territories, 2nd generation	**1.54 (1.12–2.11)**	1.37 (0.94–2.00)	1.30 (0.89–1.89)	1.21 (0.83–1.77)	**1.50 (1.09–2.06)**	1.22 (0.83–1.80)	1.18 (0.80–1.73)	1.11 (0.75–1.63)
Europe, 2nd generation	**1.17 (1.07–1.26)**	**1.15 (1.03–1.28)**	**1.12 (1.01–1.25)**	1.10 (0.99–1.23)	**1.15 (1.07–1.25)**	1.10 (0.98–1.22)	1.08 (0.96–1.20)	1.06 (0.95–1.18)
North Africa, 2nd generation	**1.47 (1.29–1.67)**	**1.25 (1.08–1.45)**	**1.19 (1.03–1.38)**	1.13 (0.97–1.32)	**1.45 (1.27–1.66)**	**1.20 (1.03–1.39)**	1.14 (0.98–1.33)	1.08 (0.93–1.27)
Sub-Saharan Africa, 2nd generation	**1.76 (1.04–2.97)**	**1.78 (1.10–2.87)**	**1.67 (1.02–2.71)**	1.57 (0.96–2.57)	1.69 (0.99–2.85)	1.45 (0.88–2.40)	1.39 (0.84–2.29)	1.30 (0.78–2.17)
Asia, 2nd generation	**1.49 (1.05–2.12)**	1.08 (0.71–1.64)	1.02 (0.67–1.56)	0.97 (0.63–1.48)	**1.47 (1.04–2.09)**	1.05 (0.68–1.61)	0.99 (0.64–1.54)	0.93 (0.60–1.45)
Other, 2nd generation	**1.69 (1.23–2.32)**	1.30 (0.93–1.82)	1.21 (0.86–1.70)	1.19 (0.84–1.67)	**1.62 (1.18–2.22)**	1.08 (0.76–1.53)	1.02 (0.72–1.44)	1.00 (0.70–1.42)

Bolded terms indicate statistically significant estimates (p < 0.05)

#### Association between immigration background and experiencing ACEs, adjusting for age and sex

We observed higher odds of experiencing any ACEs across all groups of participants (vs. native French) except 1st generation participants from Asia with AORs ranging from 1.25 (Europe, 2nd generation) to 2.21 (overseas territories, 1st generation) (Table 3). Generally, 1st and 2nd generation participants have increased odds of experiencing individual ACEs compared to the native French, except for 1st generation participants from NA, SSA, and Asia who had lower odds of having a household member with mental illness or alcohol or drug abuse (Supplemental Table 2). These associations were generally comparable when stratifying by sex.

#### Association between experiencing ACEs or the ACE count and depressive symptoms, adjusting for age and sex

Experiencing any ACE was also associated with depressive symptoms (AOR = 1.96, 95%CI: 1.89, 2.03), as was each ACE count (ACE = 1: AOR = 1.35, 95%CI: 1.28, 1.41; ACE = 2: AOR = 1.74, 95%CI: 1.66, 1.83; ACE = 3: AOR = 2.27, 95%CI: 2.15, 2.39; ACE = 4+: AOR = 3.31, 95%CI: 3.16, 3.47).

#### Association between immigration background and depressive symptoms, adjusting for experiencing ACEs or the ACE count, age, and sex

After adjusting for experiencing any ACE, associations between immigration background and depressive symptoms were attenuated between 2% and 12% with AOR ranging from 1.07 (Europe, 1 st generation) to 1.89 (North Africa, 1 st generation) (Table 3). Using the ACE count in the model attenuates associations between 0% and 21% with AOR ranging from 1.01 (Europe, 1 st generation) to 1.86 (North Africa, 1st generation), (Table 3).

### Associations after adjusting for all covariates

When adjusting for all covariates, experiencing any ACE was still associated with depressive symptoms (AOR = 1.90, 95%CI: 1.83, 1.98), as was each ACE count (ACE = 1: AOR = 1.33, 95%CI: 1.27, 1.39; ACE = 2: AOR = 1.72, 95%CI: 1.64, 1.81; ACE = 3: AOR = 2.21, 95%CI: 2.09, 2.33; ACE = 4+: AOR = 3.06, 95%CI: 2.92, 3.21). When adjusting for all covariates and not the ACEs count (Table 3), compared to the native French, there were higher odds of depressive symptoms for 1st generation adults from overseas territories and NA, and 2nd generation adults with ≥ 1 parent from Europe, NA, and SSA. After adjusting for the ACE count, these associations were attenuated between 1.0% and 15.6%, compared to 1.0%−8.6% for experiencing any ACE, and only remained significant for 1st generation adults from NA (AOR = 1.52, 95%CI: 1.29, 1.79). Overall, the findings and associations were consistent with analyses based on complete cases (Supplemental Table 3) and were generally comparable when stratified by sex with some groups showing minor differences.

### Path analysis results

The PA model estimated significant mediation effects of ACEs across all immigration backgrounds except immigrants from Asia, ranging between 0.03 and 0.10 in the minimally adjusted model, and between 0.03 and 0.09 in the fully adjusted model (Table 4). By generation, the indirect effects estimated were 0.06 and 0.04 for 1 st and 2nd generation respectively in the minimally adjusted model, and 0.05 and 0.04 for 1 st and 2nd generation in the fully adjusted model (Table 4).

**Table 4:** Estimation of causal mediation effects measured from path analysis of associations of immigration background, adverse childhood experiences, and depressive symptoms in adulthood in the CONSTANCES study, using imputed data (analyses adjusted for age and sex (*), or for age, sex, education, family average net monthly income, marital status, employment situation, and occupational grade (**)). Bolded values indicate statistically significant terms (*p* < 0.05)

Immigration Background	Indirect effects of experiencing any ACE (standard error)*	Indirect effects of experiencing any ACE (standard error)**
**Reference: native French (3rd + generation)**		
Overseas territories, 1 st generation	**0.10 (0.012)**	**0.10 (0.011)**
Europe, 1 st generation	**0.04 (0.006)**	**0.04 (0.006)**
North Africa, 1 st generation	**0.05 (0.011)**	**0.05 (0.011)**
Sub-Saharan Africa, 1 st generation	**0.09 (0.014)**	**0.08 (0.014)**
Asia, 1 st generation	0.03 (0.014)	0.03 (0.014)
Other, 1 st generation	**0.08 (0.011)**	**0.08 (0.011)**
Overseas territories, 2nd generation	**0.07 (0.013)**	**0.07 (0.012)**
Europe, 2nd generation	**0.03 (0.004)**	**0.03 (0.004)**
North Africa, 2nd generation	**0.05 (0.006)**	**0.05 (0.006)**
Sub-Saharan Africa, 2nd generation	**0.09 (0.019)**	**0.08 (0.019)**
Asia, 2nd generation	**0.05 (0.014)**	**0.04 (0.013)**
Other, 2nd generation	**0.07 (0.012)**	**0.07 (0.012)**
**By generation – reference: native French (3rd + generation)**		
1 st generation	**0.06 (0.005)**	**0.05 (0.004)**
2nd generation	**0.04 (0.003)**	**0.04 (0.003)**

## Discussion and conclusions

In a French national adult cohort, we found higher odds of experiencing ACEs and depressive symptoms in non-native participants across diverse geographic origins compared to the native French. The results regarding depressive symptoms are consistent with previous research [10, 11] and suggests that in France, increased prevalence of depression is not unique to the geographic origin, although the magnitudes of the associations vary and cultural and sociodemographic factors may play a role for certain groups. ACEs appear to have both a threshold and dose-response relationship with depressive symptoms in adulthood – specifically, experiencing any ACEs is associated with depressive symptoms, and increasing counts of ACEs show stronger associations with depressive symptoms – although these relationships need to be tested more thoroughly [44]. Associations of immigration background with depressive symptoms were partially or fully mediated by ACEs. Through path analysis, we find evidence of a potential mediating effect of ACEs, albeit generally small according to certain criteria [45], but still contributory and statistically significant.

### Study findings and context

ACEs may be associated with depression later in life through various pathways. As there is a comprehensive set of ACEs included in this study, multiple ACEs may influence the observed associations and estimated mediation effects across immigration backgrounds. Particularly in this study, financial difficulties, different types of abuse, and parental separation/death generally showed high prevalence in non-native individuals. Furthermore, it may be that ACEs influence depressive symptoms through sociodemographic factors later in life including income, education, and employment. This area of inquiry can be tested through running additional mediation analyses with corresponding models to test potential causal relationships between immigration background, ACEs, depressive symptoms, and other factors that may lie on the causal pathway between ACEs and depressive symptoms. The mediating effect of ACEs was significant for both 1st and 2nd generation adults. For 2nd generation adults, ACEs were likely largely experienced in France. Contrastingly, for 1 st generation adults, ACEs could have been experienced in the country of origin or France, depending on how long since they had immigrated to France. In this study, the average age when immigrants moved to France was 18.4 years (SD = 13). Furthermore, increased levels of ACEs among 2nd generation participants may be driven by intergenerational transmission of ACEs – essentially, parents’ experiences with ACEs influence transmission of ACEs in subsequent generations [22, 46]. While we do have data on age at immigration for immigrants, we do not have the age at which all individual ACEs occurred, so it is not possible to fully establish the temporality of some measures. However, our exposure is immigration background (a combination of geographic area of origin and immigration generation) which theoretically captures pre-, peri-, and post-migration experiences and serves more as a proxy of ethnicity/ancestry-related life experiences or being an immigrant/child of an immigrant, and altogether precedes ACEs, rather than the actual immigration experience.

There is no previous research evaluating the mediating effect of ACEs on the association between immigration background and depression in France, and limited research in other countries for different health outcomes [47, 48]. The contribution of adversity to increased likelihood of psychiatric conditions in immigrants has been reported for psychosis [49]. Studies in France have found associations between immigration generation of different geographic origins and worse psychiatric health [10, 11], but have not examined associations with ACEs. Previously reported estimates of ACEs in France have varied due to different measurement methods and study populations – including differences in individual ACEs measured and included in cumulative measures of ACE prevalence, and measurement in different, nonrepresentative samples (non-clinical populations recruited from social networks, college students only, older adults only, pregnant women, geographically specific samples) [16, 26–28, 50, 51] – but our study provides comprehensive, recent estimates of individual and cumulative ACEs in a national, diverse, large sample of French adults. We also examined additional factors beyond ACEs that could potentially influence depressive symptoms among different immigration backgrounds. Our associations are generally comparable by sex, with some differences. The similarity highlights that there may be specific risk factors in France for experiencing ACEs and depression in groups of immigrants and their descendants by sex, but an overall comparable burden producing comparable outcomes. High prevalence estimates of lower income, lower occupational grade, and higher unemployment were generally observed across 1 st and 2nd generation groups, which suggests the importance of labor market conditions as a contributor to depression, which has been reported before among second generation individuals in France [4]. Findings related to education level and marital/relationship status also depend on immigration background. In the context of immigration, sociodemographic factors may function as both confounders and mediators and be on the causal pathway between ACEs and depression in adulthood. The associations with immigration background and depressive symptoms that were mediated, fully or partially, by experiencing ACEs were also attenuated by sociodemographic factors. Taken together, financial difficulties early in life, that could be interrelated with other ACEs, or later in life related to employment, occupational grade, and income could represent an important, addressable risk factor of depression in the non-native French population. As the mediating effect of ACEs is relatively small, addressing other sociodemographic factors in addition to ACEs is important to ameliorate the risk of depression later in life in immigrants and descendants. Studying this research topic for mental health outcomes in adolescence and young adulthood is also an important area for future research.

First generation CONSTANCES study participants represent heterogenous groups that arrived in mainland, post-World War II France. Initially for several decades of this period, immigration was driven by economic reconstruction, with some expectations that immigrants, particularly those who arrived from Africa around the 1950–1960s would return home in the short-term after having earned some income in France [52]. In the mid-1970s, more restrictions on immigration were placed, especially from low-income countries, concurrent with ongoing economic crises and recessions [52]. Still, the prospects of many immigrants changed, and some had the desire to live in France indefinitely, even bringing their families. From the 1970s, family reunification further changed the demographic structure of immigration and the growth of migration flows from 2000 onwards [53]. Additionally, the establishment of the Schengen area and expansion of the European Union (EU) led to increased migration from other EU countries [4]. After controlling for sociodemographic characteristics, associations between immigration background and depressive symptoms were all partially or fully attenuated, with further attenuation after measures of ACEs were included, and only remained statistically significant for 1st generation participants from NA. It is unclear why the association remains for immigrants from NA, whilst not for others. Experiences related to discrimination [34], integration and unique migration-related histories [54] not accounted for in the ACE count and study variables may explain this finding. Additional factors including mental illness stigma, religious views, and other sociocultural factors in the North African context that might impact, or delay help-seeking could also be relevant to these findings [55–58] – although, they could potentially be expected to reduce the prevalence of depressive symptoms.

### Study limitations, strengths, and conclusions

There are several limitations to our study. First, there are multiple assumptions behind a mediation analysis and a causal interpretation of the results [59] which may not be fully satisfied by the data collection and study design. One of the key assumptions behind a causal mediation analysis is there should be no unmeasured confounding. Potential unmeasured confounders not collected in the CONSTANCES data may not be fully controlled for in the relationship between the exposure-outcome, exposure-mediator, and mediator-outcome relationships. Examples of such factors include social isolation and discrimination, and those at the neighborhood-level such as immigrant density [60, 61]. Another assumption behind a mediation analysis is that there should be no exposure-induced mediator-outcome confounding [59]. There could be effects of the exposure that confounds the mediator-outcome relationship through unmeasured confounders including bullying [62, 63], self-esteem [64], biological factors [65–67], and other adult lifestyle factors. However, the robustness of findings across varying sensitivity analyses conducted in this study (i.e., minimally and fully adjusted models, and by specific geographic origins and generation) support the conclusions of this study.

Second, as mentioned, the CONSTANCES target population is restricted to individuals affiliated to the general scheme of the national healthcare system and knowledge of the French language, with significant loss to follow-up. Consequently, individuals who lack access to healthcare or do not speak or understand French well, including undocumented migrants, are not represented in the cohort. The proportion and demographics of 1st and 2nd generation individuals in this dataset differ from French Census-based assessments and the population at large [4]. For instance, the CONSTANCES cohort consists of older adults on average, and contains a lower proportion of immigrants compared to previous Census population estimates [1]. However, the proportion of second-generation participants is consistent with estimates from French census data [4]. Overall, the large, diverse sample is representative of a majority immigrants living in France. Third, we are limited by the resolution of data regarding participants’ grandparental geographic origin collected in the CONSTANCES cohort. Thus, we are unable to disaggregate the native French group further, like the 1st and 2nd generations. Furthermore, the sizes of some subgroups are small, which limits the statistical power to detect significant effects. We recognize individuals born in France to one immigrant parent may share some characteristics with both those with two immigrant parents and those with two native-born parents, due to potential differences in cultural challenges, experiences of social isolation and integration, and other factors. However, given sample size considerations and at least the presence of a partial experience of a different cultural background, we have grouped participants based on the standard practice and definition [29, 34, 68].

Fourth, this study focuses on ACEs that were collected in the CONSTANCES study. However, we are unable to include other unmeasured childhood or adult experiences, such as childhood neglect or discrimination. However, some of the ACEs included in this study covered some experiences which could be classified as neglect, such as loss and incarceration of a parent (e.g., emotional neglect) and family financial difficulties (material neglect), despite not directly focusing on experiences of neglect in the study. Furthermore, not all ACEs are experienced equally, i.e., with the same intensity or life impact. Certain ACEs may be more traumatic experiences compared to other ACEs (e.g., experiencing physical or sexual abuse compared to verbal abuse). Furthermore, individuals may experience ACEs differently – for instance, parental separation during childhood may be distressing to one individual, but minimally impactful to another individual. Nevertheless, the set of ACEs included in this study are comprehensive, and as certain types of ACEs are interrelated [69], ACEs included in the study could serve as a proxy for other unmeasured ACEs. There is a risk of recall bias with the measurement of ACEs in this study as they were collected retrospectively. Previous research has suggested that prospective vs. retrospective [70–72] or self- vs. agency-reported ACEs can vary [73]. However, the same body of research has also demonstrated associations between ACEs and the health outcomes studied, regardless of the method of measurement [70–73].

Fifth, the data for this study were not weighted to be nationally representative for France, with a significant number of participants also not completing the follow-up questionnaire for the study year used. The loss to follow-up did also vary by exposure category (range by immigration background: 39–71%), with higher loss to follow-up among immigrants compared to 2nd-generation and native participants (chi-square p-value < 0.05). Thus, the representativeness and generalizability of the study need to be interpreted with caution. Sixth, the outcome in this study is not necessarily considered a rare outcome (> 10% in the sample) but can still be appropriately modeled with logistic regression modeling in mediation analyses. Furthermore, the method utilized in this approach is in fact conservative and may underestimate the measured indirect effect.

Despite these limitations, our study also has several strengths. First, the associations are still theoretically sound and based on a large cohort size with sufficient statistical power to examine associations across varying geographic origins. Second, there is a low risk of collider bias, where depression in adulthood is also linked to report of experiencing ACEs which subsequently produces a distorted association and estimate. To our knowledge, there is no previous study or evidence that directly shows that depressive symptoms in adulthood are linked to differentially experiencing or reporting ACEs, even if ACEs would be easier to recall. Previous research regarding the relationship between depression status and autobiographical memory-experience is mixed, depends on multiple factors and is lacking in immigrants and their descendants. Some research suggests that depressed individuals may have a hard time remembering past events [74], and other research suggests that memory biases in emotionally disturbed people may be more related to the specificity of memories (i.e. summarize categories of events rather than single episodes) [75] and emotions [76]. Using path analysis for a mediation analysis provides methodological advantages [41], and our findings suggest that in France, experiencing ACEs may have a mediating effect on depressive symptoms among most groups of immigrants and descendants.

## Supplementary Information

Supplementary Material 1

## Data Availability

The data used in this study cannot be made publicly available according to French data protection laws. Any questions about access to the data can be addressed to the corresponding author.

## Abbreviations

ACEs: Adverse childhood experiences
AOR: Adjusted odds ratio
CI: confidence interval
