# Supplementary material for: Associations between immigration background, adverse childhood experiences, and depressive symptoms in adulthood in immigrants and descendants of immigrants in France: a mediation analysis
Source: Ann Gen Psychiatry. 2026 Feb 17;25:14. doi: 10.1186/s12991-025-00612-7 (PMC12931066; doi:10.1186/s12991-025-00612-7)
Supplement: Supplementary file 1 — Supplementary Material 1 [file 12991_2025_612_MOESM1_ESM.docx]

**Supplementary Online-Only Material**

**Supplemental Methods**

***Data Sources***

The target population of CONSTANCES is restricted to individuals affiliated to the general scheme of the national healthcare system (covering >85% of the French population in 21 selected departments across metropolitan France). Agricultural and self-employed workers are generally excluded from the study although some were still included during recruitment. Eligible individuals were selected using a random sampling scheme stratified on place of residence, age, sex, occupation, and socio-economic status to be representative of the target population.

Recruitment of CONSTANCES participants was conducted in French through Health Screening Centers (HSCs) with previous research experience that geographically represent principal regions of France.[1] Informed consent was obtained from all participants. For study inclusion, eligible people receive an invitation to complete questionnaires and receive a comprehensive health examination at their HSC. Participants are then followed-up through an annual self-administered questionnaire (paper or online), completed at home. This study used data collected at inclusion and the 2020 follow-up.[2] For the CONSTANCES study, the average response rate of annual follow-up questionnaires was >60% with variations depending on the year.[2]

***Exposure***

Some participants (n=338) reported parents born in different regions outside of mainland/metropolitan France, in which case geographic origin was contextually assigned. Specifically, having a parent from French overseas territories would take first precedence because the parent could be a French citizen from birth; having a parent from Europe would be prioritized next as the parent could be a European Union citizen and have closer family ties; followed by having a parent from NA, SSA, Asia, and “Other” origin, in order, given factors including proximity to France, and closeness of potential family ties.

***Covariates***

Age at study inclusion and sex (male vs. female) were included in the analysis. Educational level at study inclusion was defined as the highest diploma received (≤secondary school vs. >secondary school). Income was defined as the household's average net monthly income – the sum of incomes of all persons contributing to the household's expenses, or the individual’s income if they live alone (categorized as < 2100 € or ≥ 2100 € per month, with the cut-off based on the estimated average net monthly household income during the recruitment period[3, 4]). Marital status was categorized as married or in a civil partnership vs. not (i.e., never married, separated, divorced, or a widow or widower). Employment was categorized as “has a job (including sick, unpaid, maternity/paternity/adoption/parental leave)” or “retired or no longer in business” vs. any other employment situation (“Unemployed or job seeker”, “in training” as a pupil, student, trainee, apprentice, etc., “does not work for health reasons such as long-term illness or disability, “no professional activity”, or “other” category). Occupational grade (or held for the longest time if the participant was not currently working) was dichotomized as participants who are an “executive or higher intellectual professional (engineer, physician, etc.)”, “intermediate professional (teacher, nurse, social worker, technician, foreman, etc.)”, or “craftsperson, shopkeeper, business owner, etc.” (i.e., ≥ intermediate professional or business owner), vs. those who are an “employee (office or commercial employee, child minder, duty officer, etc.)”, “farmer or farmer’s spouse”, “manual worker”, individual who “has never worked”, or “other” occupational grade (i.e., < intermediate professional or business owner). For a separate analysis involving 1^st^ generation participants, we measured the time lived in France, dichotomized as <10 years or ≥10 years.

***Statistical Analysis***

Between 1.5%-6.3% of cases had missing data for some study variables. Prevalence and model parameter estimates were calculated based on complete cases using the SAS FREQ and LOGISTIC procedures. Multiple imputation was used to handle missing data and performed using fully conditional specification methods implemented in R (Multivariate Imputation by Chained Equations package; R software version 3.6).[5] The variables used in the multiple imputation procedure included the geographic origin, nationality, ACEs, and sociodemographic information collected in CONSTANCES. Ten imputed datasets were created and used for model fitting. Statistical models were fitted, and parameter estimates were summarized using multiple imputation methods implemented in SAS.

***Ethical Approval***

This project received ethical approval from the Sorbonne Université comité d’éthique de la recherché (Protocol number: "CER-2023-Kasra-NGACEMSF”) and was carried out within the framework of the CONSTANCES study. The CONSTANCES cohort was authorized by the French personal data privacy authority (CNIL #910486) and was approved by the Institutional Review Board of French Institute of Health (INSERM, IRB #01-011 and 21-842).

**Supplemental Results**

Over half of participants were female (54.9%) with a sample mean age of 48.8 years (standard deviation = 13.1 years). Most participants reported having a family average net monthly income of ≥€2100 (76.9%); having a degree higher than a French secondary school diploma (62.8%); being married or in a civil partnership (62.6%); having a job, being retired or no longer in business (88.8%); or having an occupational grade ≥ an intermediate professional or craftsperson, shopkeeper, or business owner (63.7%).

Supplemental Table 1 reports the prevalence of study variables by immigration background. Compared to the native French, groups of 1^st^ and 2^nd^ generation participants had higher prevalence of having a lower average family net monthly income. Furthermore, some groups of 1^st^ generation participants had higher prevalence of having a lower education level compared to native French, specifically those from overseas territories (49.3% vs. 36.6%;), NA (44.8% vs. 36.6%), and SSA (47.8% vs. 36.6%). Contrastingly, 1^st^ generation participants from Europe (33.1% vs. 36.6%), Asia (24.9% vs. 36.6%) and “Other” origin (21.1% vs. 36.6%) had lower prevalence of having a lower education level, as did 2^nd^ generation participants from NA (28.1% vs. 36.6%) and Asia (26.3% vs. 36.6%). Groups of 1^st^ and 2^nd^ generation participants generally had higher prevalence of not being married or in a civil partnership, not having a job, not being retired or in business, or having a lower occupational grade. Among 1^st^ generation participants, time in France was not associated with depressive symptoms (AOR=1.01, 95%CI: 0.86, 1.17; p>0.05).

1. Zins, M., M. Goldberg, and C. team, *The French CONSTANCES population-based cohort: design, inclusion and follow-up.* Eur J Epidemiol, 2015. **30**(12): p. 1317-28.

2. Henny, J., et al., *The CONSTANCES Cohort Biobank: An Open Tool for Research in Epidemiology and Prevention of Diseases.* Front Public Health, 2020. **8**: p. 605133.

3. *L'Institut national de la statistique et des études économiques. Household income and wealth.* 2016: France.

4. *OECD Better Life Index: France*. 2024; Available from: <https://www.oecdbetterlifeindex.org/countries/france/>.

5. Azur, M.J., et al., *Multiple imputation by chained equations: what is it and how does it work?* Int J Methods Psychiatr Res, 2011. **20**(1): p. 40-9.

**Supplemental Table 1.** Prevalence and mean estimates of sociodemographic factors, depression symptoms in adulthood, and experiencing adverse childhood experiences (ACEs) by immigration background among participants who completed the 2020 follow-up questionnaire of the CONSTANCES study (analysis based on imputed data).

|  | 1^st^ Generation | | | | | | 2^nd^ Generation | | | | | |  |
| --- | --- | --- | --- | --- | --- | --- | --- | --- | --- | --- | --- | --- | --- |
| Variable / Immigration Background | Overseas Territories | Europe | North Africa | Sub-Saharan Africa | Asia | Other | Overseas Territories | Europe | North Africa | Sub-Saharan Africa | Asia | Other | Native French |
| Age at study inclusion (mean years) | 47.1 | 49.2 | 52.6 | 46.1 | 44.7 | 47.4 | 41.0 | 49.7 | 44.1 | 39.4 | 42.2 | 45.6 | 48.8 |
| Sex |  |  |  |  |  |  |  |  |  |  |  |  |  |
| Male | 42.7% | 43.4% | 59.4% | 58.4% | 40.0% | 38.0% | 41.9% | 46.2% | 45.1% | 45.1% | 38.7% | 40.4% | 45.1% |
| Female | 57.3% | 56.6% | 40.6% | 41.6% | 60.0% | 62.0% | 58.1% | 53.8% | 54.9% | 54.9% | 61.3% | 59.6% | 54.9% |
| Family average net monthly income |  |  |  |  |  |  |  |  |  |  |  |  |  |
| <€2100 per month | 34.7% | 22.9% | 42.7% | 48.1% | 33.2% | 28.8% | 29.3% | 19.0% | 20.4% | 30.1% | 22.5% | 28.2% | 17.3% |
| ≥€2100 per month | 65.3% | 77.1% | 57.3% | 51.9% | 66.8% | 71.2% | 70.7% | 81.0% | 79.6% | 69.9% | 77.5% | 71.8% | 82.7% |
| Highest level of education |  |  |  |  |  |  |  |  |  |  |  |  |  |
| Secondary school or lower | 49.3% | 33.1% | 44.8% | 47.8% | 24.9% | 21.1% | 30.8% | 38.1% | 28.1% | 27.6% | 26.3% | 35.6% | 36.6% |
| Higher than secondary school | 50.7% | 66.9% | 55.2% | 52.2% | 75.1% | 79.9% | 69.2% | 61.9% | 71.9% | 72.4% | 73.7% | 64.4% | 63.4% |
| Marital status |  |  |  |  |  |  |  |  |  |  |  |  |  |
| Not married | 51.4% | 36.2% | 34.2% | 43.4% | 33.9% | 35.1% | 51.1% | 38.6% | 44.0% | 53.7% | 52.4% | 49.6% | 35.6% |
| Married or in a civil partnership | 48.6% | 63.8% | 65.8% | 56.6% | 66.1% | 64.9% | 48.9% | 61.4% | 56.0% | 46.3% | 47.6% | 50.4% | 64.4% |
| Employment |  |  |  |  |  |  |  |  |  |  |  |  |  |
| Has a job or retired or no longer in business | 86.8% | 87.5% | 80.8% | 74.9% | 74.7% | 79.1% | 84.7% | 90.0% | 87.3% | 78.6% | 86.6% | 84.8% | 91.6% |
| Other employment situation | 13.2% | 12.5% | 19.2% | 25.1% | 25.3% | 20.9% | 15.3% | 10.0% | 12.7% | 21.4% | 13.4% | 15.2% | 8.4% |
| Occupational grade |  |  |  |  |  |  |  |  |  |  |  |  |  |
| ≥ Intermediate professional, business owner | 50.7% | 61.7% | 48.2% | 36.9% | 51.1% | 62.6% | 57.8% | 65.2% | 68.0% | 62.9% | 66.3% | 60.6% | 67.2% |
| < Intermediate professional, business owner | 49.3% | 38.3% | 51.8% | 63.1% | 48.9% | 37.4% | 42.2% | 34.8% | 32.0% | 37.1% | 33.7% | 39.4% | 32.8% |
| Depression symptoms | 27.8% | 20.7% | 29.4% | 26.6% | 24.6% | 23.5% | 25.3% | 20.8% | 21.6% | 27.9% | 22.2% | 24.0% | 19.0% |
| Any ACE | 81.6% | 72.3% | 72.9% | 78.9% | 70.9% | 78.7% | 79.1% | 70.7% | 74.8% | 81.1% | 74.6% | 77.4% | 66.1% |
| ACE count |  |  |  |  |  |  |  |  |  |  |  |  |  |
| 0 | 18.4% | 27.7% | 27.1% | 21.0% | 29.1% | 21.3% | 20.9% | 29.3% | 25.2% | 18.9% | 25.4% | 22.6% | 33.9% |
| 1 | 20.0% | 19.9% | 22.5% | 23.1% | 22.9% | 17.3% | 19.4% | 22.2% | 20.6% | 19.5% | 20.6% | 24.6% | 23.6% |
| 2 | 20.4% | 19.4% | 20.3% | 18.5% | 22.1% | 19.0% | 19.1% | 19.2% | 19.6% | 24.0% | 19.2% | 16.9% | 18.0% |
| 3 | 16.3% | 15.0% | 14.6% | 15.3% | 13.7% | 19.8% | 16.0% | 13.5% | 14.9% | 9.8% | 14.0% | 15.1% | 11.8% |
| 4+ | 24.9% | 18.0% | 15.5% | 22.1% | 12.1% | 22.6% | 24.6% | 15.8% | 19.8% | 27.8% | 20.8% | 20.8% | 12.7% |
| Adverse childhood experiences |  |  |  |  |  |  |  |  |  |  |  |  |  |
| Parental separation or death | 34.4% | 19.0% | 19.3% | 34.2% | 20.9% | 32.7% | 34.6% | 18.6% | 24.2% | 36.4% | 21.6% | 32.9% | 17.1% |
| Family financial difficulties | 34.9% | 24.2% | 33.3% | 31.2% | 26.4% | 29.5% | 27.8% | 25.0% | 27.1% | 29.5% | 28.9% | 26.1% | 19.1% |
| Household member with mental illness | 19.7% | 18.6% | 13.1% | 9.6% | 12.3% | 20.2% | 24.0% | 21.2% | 26.3% | 26.9% | 25.0% | 22.8% | 19.6% |
| Household member with alcohol or drug abuse | 25.5% | 21.4% | 12.2% | 17.1% | 11.7% | 24.2% | 24.2% | 20.3% | 20.2% | 19.6% | 18.9% | 22.9% | 18.4% |
| Household member incarcerated | 2.3% | 1.6% | 2.1% | 4.3% | 2.2% | 2.3% | 2.5% | 1.4% | 2.6% | 4.8% | 1.3% | 3.6% | 1.0% |
| Verbal abuse from household member | 52.5% | 47.6% | 48.4% | 53.8% | 44.4% | 49.7% | 51.2% | 43.7% | 49.3% | 54.8% | 51.1% | 44.8% | 39.1% |
| Sexual abuse from household member | 18.5% | 15.0% | 14.5% | 15.4% | 14.5% | 21.0% | 16.6% | 13.5% | 15.3% | 20.2% | 14.7% | 16.7% | 12.1% |
| Witnessing or experiencing household physical violence | 44.1% | 39.8% | 36.6% | 43.7% | 35.2% | 41.8% | 42.6% | 31.7% | 35.8% | 40.9% | 38.3% | 35.1% | 28.2% |

**Supplemental Table 2.** Associations between immigration background and individual adverse childhood experiences among participants who completed the 2020 follow-up questionnaire of the CONSTANCES study (France, 2012-present, logistic regression models adjusted for age and sex; adjusted odds ratio [AORs], 95% confidence intervals [95% CI]).

|  | AOR (95% CI) | | | | | | |  |
| --- | --- | --- | --- | --- | --- | --- | --- | --- |
| Immigration Background | Parental separation or death | Family financial difficulties | Household member with mental illness | Household member with alcohol or drug abuse | Household member incarcerated | Verbal abuse from household member | Sexual abuse from household member | Witnessing or experiencing household physical violence |
| **Reference: native French (3rd+ generation)** |  |  |  |  |  |  |  |  |
| **Overall** |  |  |  |  |  |  |  |  |
| Overseas territories, 1^st^ generation | **2.43 (2.05-2.87)** | **2.28 (1.91-2.71)** | 0.94 (0.75-1.17) | **1.49 (1.25-1.79)** | **2.10 (1.21-3.63)** | **1.67 (1.41-1.97)** | **1.65 (1.32-2.05)** | **1.98 (1.69-2.33)** |
| Europe, 1^st^ generation | **1.16 (1.04-1.28)** | **1.34 (1.22-1.48)** | 0.94 (0.85-1.05) | **1.21 (1.10-1.34)** | **1.60 (1.17-2.19)** | **1.43 (1.32-1.55)** | **1.28 (1.14-1.43)** | **1.69 (1.56-1.83)** |
| North Africa, 1^st^ generation | 1.17 (0.97-1.42) | **2.20 (1.90-2.56)** | **0.66 (0.53-0.82)** | **0.64 (0.51-0.79)** | **2.18 (1.34-3.55)** | **1.46 (1.26-1.70)** | **1.46 (1.19-1.79)** | **1.49 (1.29-1.72)** |
| Sub-Saharan Africa, 1^st^ generation | **2.46 (2.03-2.98)** | **2.01 (1.63-2.48)** | **0.44 (0.32-0.62)** | 0.93 (0.73-1.20) | **4.30 (2.73-6.77)** | **1.77 (1.44-2.16)** | **1.55 (1.19-2.03)** | **1.98 (1.64-2.39)** |
| Asia, 1^st^ generation | 1.20 (0.95-1.51) | **1.52 (1.21-1.89)** | **0.52 (0.38-0.71)** | **0.57 (0.43-0.75)** | **1.99 (1.05-3.75)** | 1.20 (0.99-1.45) | 1.18 (0.89-1.57) | **1.35 (1.12-1.64)** |
| Other, 1^st^ generation | **2.30 (1.96-2.69)** | **1.75 (1.48-2.07)** | 0.98 (0.80-1.19) | **1.38 (1.17-1.64)** | **2.21 (1.35-3.63)** | **1.52 (1.30-1.77)** | **1.84 (1.52-2.23)** | **1.81 (1.56-2.10)** |
| Overseas territories, 2^nd^ generation | **2.26 (1.87-2.72)** | **1.66 (1.35-2.04)** | 1.10 (0.87-1.39) | **1.35 (1.09-1.66)** | **2.11 (1.09-4.06)** | **1.50 (1.25-1.79)** | **1.43 (1.11-1.84)** | **1.82 (1.52-2.18)** |
| Europe, 2^nd^ generation | **1.12 (1.05-1.20)** | **1.41 (1.33-1.50)** | **1.13 (1.06-1.20)** | **1.14 (1.07-1.21)** | **1.42 (1.14-1.77)** | **1.22 (1.16-1.29)** | **1.15 (1.07-1.24)** | **1.19 (1.12-1.25)** |
| North Africa, 2^nd^ generation | **1.43 (1.31-1.56)** | **1.60 (1.47-1.75)** | **1.35 (1.23-1.48)** | 1.09 (0.99-1.20) | **2.34 (1.84-2.98)** | **1.44 (1.33-1.55)** | **1.34 (1.20-1.49)** | **1.39 (1.28-1.50)** |
| Sub-Saharan Africa, 2^nd^ generation | **2.37 (1.78-3.16)** | **1.83 (1.32-2.54)** | 1.27 (0.92-1.73) | 1.02 (0.72-1.45) | **4.04 (2.14-7.66)** | **1.70 (1.29-2.25)** | **1.91 (1.34-2.72)** | **1.69 (1.27-2.24)** |
| Asia, 2^nd^ generation | 1.18 (0.94-1.48) | **1.73 (1.40-2.13)** | 1.17 (0.94-1.45) | 0.98 (0.76-1.25) | 1.06 (0.42-2.65) | **1.51 (1.25-1.82)** | 1.19 (0.91-1.56) | **1.53 (1.26-1.85)** |
| Other, 2^nd^ generation | **2.24 (1.88-2.68)** | **1.49 (1.23-1.81)** | 1.11 (0.90-1.37) | **1.28 (1.05-1.56)** | **3.34 (2.14-5.20)** | **1.22 (1.02-1.46)** | **1.42 (1.12-1.80)** | **1.35 (1.14-1.61)** |
| **Stratification by Sex** |  |  |  |  |  |  |  |  |
| Female |  |  |  |  |  |  |  |  |
| Overseas territories, 1^st^ generation | **2.51 (2.01-3.12)** | **2.16 (1.72-2.71)** | 1.02 (0.77-1.36) | **1.59 (1.27-2.00)** | **2.30 (1.18-4.47)** | **1.78 (1.44-2.21)** | **1.69 (1.32-2.17)** | **2.17 (1.75-2.69)** |
| Europe, 1^st^ generation | **1.30 (1.14-1.47)** | **1.30 (1.15-1.47)** | 0.95 (0.83-1.09) | **1.27 (1.13-1.44)** | 1.37 (0.89-2.11) | **1.46 (1.31-1.63)** | **1.24 (1.09-1.42)** | **1.68 (1.51-1.86)** |
| North Africa, 1^st^ generation | 1.14 (0.86-1.52) | **1.90 (1.49-2.41)** | **0.71 (0.52-0.96)** | **0.73 (0.54-0.99)** | 1.47 (0.59-3.66) | **1.85 (1.48-2.31)** | 1.26 (0.94-1.69) | **1.67 (1.32-2.10)** |
| Sub-Saharan Africa, 1^st^ generation | **2.48 (1.84-3.35)** | **1.82 (1.33-2.49)** | **0.52 (0.33-0.83)** | 1.09 (0.77-1.55) | **4.17 (2.13-8.19)** | **1.70 (1.26-2.28)** | **1.67 (1.17-2.37)** | **1.76 (1.31-2.36)** |
| Asia, 1^st^ generation | 1.10 (0.82-1.48) | 1.31 (0.98-1.75) | **0.53 (0.36-0.77)** | **0.62 (0.44-0.87)** | 1.77 (0.78-4.00) | 1.04 (0.81-1.33) | 1.11 (0.80-1.53) | 1.11 (0.86-1.43) |
| Other, 1^st^ generation | **2.36 (1.93-2.88)** | **1.67 (1.35-2.06)** | 0.89 (0.70-1.13) | **1.31 (1.05-1.63)** | 1.22 (0.55-2.72) | **1.44 (1.18-1.75)** | **1.75 (1.41-2.18)** | **1.56 (1.29-1.89)** |
| Overseas territories, 2^nd^ generation | **2.48 (1.94-3.17)** | **1.82 (1.41-2.35)** | 1.15 (0.86-1.53) | **1.42 (1.09-1.85)** | **2.61 (1.31-5.19)** | **1.53 (1.21-1.93)** | **1.37 (1.02-1.82)** | **1.80 (1.42-2.28)** |
| Europe, 2^nd^ generation | **1.12 (1.02-1.22)** | **1.45 (1.34-1.58)** | **1.17 (1.07-1.27)** | **1.25 (1.15-1.36)** | **1.47 (1.11-1.94)** | **1.28 (1.19-1.38)** | **1.21 (1.11-1.32)** | **1.21 (1.13-1.30)** |
| North Africa, 2^nd^ generation | **1.49 (1.32-1.67)** | **1.58 (1.41-1.76)** | **1.36 (1.21-1.54)** | 1.05 (0.93-1.19) | **2.20 (1.60-3.02)** | **1.47 (1.32-1.63)** | **1.31 (1.16-1.48)** | **1.39 (1.25-1.54)** |
| Sub-Saharan Africa, 2^nd^ generation | **2.58 (1.77-3.76)** | **1.96 (1.29-2.98)** | 1.47 (0.99-2.17) | 1.13 (0.72-1.76) | **3.79 (1.65-8.68)** | **1.72 (1.18-2.51)** | **2.02 (1.33-3.05)** | **1.89 (1.30-2.75)** |
| Asia, 2^nd^ generation | 1.25 (0.94-1.65) | **1.63 (1.24-2.15)** | 1.18 (0.91-1.54) | 0.89 (0.65-1.22) | 0.88 (0.28-2.82) | **1.54 (1.21-1.97)** | 1.06 (0.77-1.45) | **1.48 (1.16-1.89)** |
| Other, 2^nd^ generation | **2.17 (1.72-2.74)** | **1.52 (1.18-1.96)** | 1.16 (0.89-1.50) | **1.35 (1.05-1.72)** | **2.77 (1.51-5.11)** | **1.39 (1.11-1.74)** | **1.44 (1.10-1.88)** | **1.37 (1.10-1.72)** |
| Male |  |  |  |  |  |  |  |  |
| Overseas territories, 1^st^ generation | **2.32 (1.79-3.02)** | **2.46 (1.87-3.23)** | 0.79 (0.55-1.14) | **1.35 (1.00-1.82)** | 1.75 (0.66-4.63) | **1.53 (1.16-2.01)** | 1.52 (0.99-2.32) | **1.75 (1.37-2.24)** |
| Europe, 1^st^ generation | 0.98 (0.83-1.15) | **1.41 (1.22-1.64)** | 0.93 (0.79-1.11) | 1.13 (0.97-1.32) | **1.97 (1.24-3.12)** | **1.40 (1.24-1.58)** | **1.38 (1.11-1.71)** | **1.70 (1.51-1.92)** |
| North Africa, 1^st^ generation | 1.20 (0.94-1.52) | **2.47 (2.03-2.99)** | **0.61 (0.45-0.82)** | **0.56 (0.41-0.76)** | **2.80 (1.56-5.04)** | **1.24 (1.02-1.50)** | **1.79 (1.32-2.43)** | **1.37 (1.14-1.66)** |
| Sub-Saharan Africa, 1^st^ generation | **2.45 (1.90-3.15)** | **2.18 (1.66-2.86)** | **0.36 (0.21-0.61)** | 0.81 (0.57-1.14) | **4.45 (2.40-8.26)** | **1.81 (1.39-2.37)** | 1.40 (0.88-2.21) | **2.15 (1.70-2.73)** |
| Asia, 1^st^ generation | 1.36 (0.95-1.96) | **1.90 (1.33-2.73)** | **0.49 (0.26-0.91)** | **0.47 (0.28-0.81)** | 2.40 (0.89-6.49) | **1.47 (1.10-1.97)** | 1.43 (0.84-2.44) | **1.81 (1.34-2.43)** |
| Other, 1^st^ generation | **2.22 (1.70-2.90)** | **1.91 (1.44-2.54)** | 1.17 (0.85-1.61) | **1.53 (1.15-2.03)** | **4.24 (2.23-8.08)** | **1.67 (1.31-2.13)** | **2.19 (1.50-3.21)** | **2.29 (1.80-2.91)** |
| Overseas territories, 2^nd^ generation | **1.96 (1.44-2.66)** | 1.41 (0.99-2.01) | 1.02 (0.70-1.50) | 1.24 (0.88-1.75) | 1.15 (0.27-4.84) | **1.46 (1.10-1.93)** | **1.63 (1.01-2.63)** | **1.85 (1.41-2.45)** |
| Europe, 2^nd^ generation | **1.13 (1.03-1.25)** | **1.36 (1.23-1.50)** | 1.07 (0.96-1.18) | 0.99 (0.90-1.10) | 1.35 (0.95-1.92) | **1.16 (1.07-1.25)** | 0.99 (0.84-1.15) | **1.16 (1.07-1.26)** |
| North Africa, 2^nd^ generation | **1.37 (1.20-1.56)** | **1.64 (1.43-1.89)** | **1.32 (1.14-1.52)** | **1.16 (1.00-1.33)** | **2.56 (1.77-3.70)** | **1.40 (1.25-1.57)** | **1.44 (1.17-1.76)** | **1.39 (1.24-1.56)** |
| Sub-Saharan Africa, 2^nd^ generation | **2.12 (1.36-3.31)** | 1.65 (0.99-2.75) | 0.98 (0.56-1.74) | 0.88 (0.50-1.55) | **4.43 (1.62-12.12)** | **1.67 (1.10-2.54)** | 1.63 (0.80-3.35) | 1.46 (0.95-2.24) |
| Asia, 2^nd^ generation | 1.08 (0.73-1.59) | **1.90 (1.34-2.68)** | 1.14 (0.77-1.70) | 1.14 (0.78-1.67) | 1.40 (0.35-5.55) | **1.46 (1.08-1.99)** | **1.73 (1.05-2.85)** | **1.60 (1.17-2.18)** |
| Other, 2^nd^ generation | **2.36 (1.79-3.12)** | 1.44 (0.99-2.09) | 1.02 (0.68-1.52) | 1.17 (0.84-1.63) | **4.37 (2.24-8.52)** | 1.01 (0.76-1.34) | 1.37 (0.85-2.21) | **1.33 (1.01-1.74)** |

**Supplemental Table 3.** Associations between immigration background, adverse childhood experiences, and depression symptoms in adulthood in the CONSTANCES study (logistic regression models adjusted for age and sex (*), or for age, sex, education, family average net monthly income, marital status, employment situation, and socio-professional category (**), adjusted odds ratio [AORs], 95% confidence intervals [95% CI]): complete cases analysis.

|  | Complete Case Analysis | | | | | | | |
| --- | --- | --- | --- | --- | --- | --- | --- | --- |
| Immigration Background | Any ACE regressed on immigration background and demographics – AOR (95% CI)* | Depression symptoms regressed on immigration background and demographics – AOR (95% CI)* | Depression symptoms regressed on immigration background, any ACE, and demographics – AOR (95% CI)* | Depression symptoms regressed on immigration background, ACE count (0-4+), demographics – AOR (95% CI)* | Any ACE regressed on immigration background and demographics – AOR (95% CI)** | Depression symptoms regressed on immigration background and demographics – AOR (95% CI)** | Depression symptoms regressed on immigration background, any ACE, and demographics (95% CI)** | Depression symptoms regressed on immigration background, ACE count (0-4+), demographics (95% CI)** |
| **Reference: native French (3rd+ generation)** |  |  |  |  |  |  |  |  |
| **Overall** |  |  |  |  |  |  |  |  |
| Overseas territories, 1^st^ generation | **2.08 (1.72-2.53)** | **1.64 (1.36-1.96)** | **1.49 (1.24-1.80)** | **1.40 (1.15-1.69)** | **1.99 (1.60-2.47)** | **1.30 (1.05-1.60)** | 1.19 (0.96-1.47) | 1.13 (0.91-1.40) |
| Europe, 1^st^ generation | **1.33 (1.22-1.45)** | 1.10 (0.99-1.21) | 1.06 (0.96-1.17) | 1.02 (0.92-1.12) | **1.33 (1.21-1.46)** | 1.00 (0.90-1.12) | 0.97 (0.87-1.09) | 0.93 (0.83-1.04) |
| North Africa, 1^st^ generation | **1.33 (1.14-1.54)** | **2.04 (1.74-2.39)** | **1.99 (1.69-2.34)** | **1.96 (1.66-2.30)** | **1.26 (1.06-1.50)** | **1.65 (1.37-1.97)** | **1.60 (1.33-1.92)** | **1.58 (1.32-1.90)** |
| Sub-Saharan Africa, 1^st^ generation | **1.66 (1.35-2.05)** | **1.65 (1.33-2.05)** | **1.54 (1.23-1.92)** | **1.46 (1.17-1.83)** | **1.52 (1.20-1.93)** | 1.18 (0.92-1.51) | 1.11 (0.86-1.43) | 1.07 (0.83-1.38) |
| Asia, 1^st^ generation | 1.11 (0.91-1.35) | **1.34 (1.07-1.68)** | **1.32 (1.05-1.65)** | **1.33 (1.06-1.66)** | 1.07 (0.86-1.34) | 1.22 (0.95-1.57) | 1.19 (0.92-1.53) | 1.21 (0.94-1.56) |
| Other, 1^st^ generation | **1.84 (1.55-2.20)** | **1.21 (1.01-1.45)** | 1.12 (0.93-1.34) | 1.03 (0.86-1.25) | **1.79 (1.47-2.18)** | 1.11 (0.91-1.37) | 1.05 (0.85-1.29) | 0.98 (0.80-1.21) |
| Overseas territories, 2^nd^ generation | **1.83 (1.47-2.28)** | **1.39 (1.12-1.71)** | **1.27 (1.02-1.57)** | 1.17 (0.94-1.46) | **1.85 (1.46-2.35)** | 1.14 (0.90-1.43) | 1.03 (0.82-1.31) | 0.96 (0.76-1.22) |
| Europe, 2^nd^ generation | **1.24 (1.17-1.31)** | **1.13 (1.06-1.21)** | **1.10 (1.03-1.18)** | **1.08 (1.01-1.15)** | **1.24 (1.17-1.32)** | **1.09 (1.02-1.17)** | 1.07 (0.99-1.15) | 1.04 (0.97-1.12) |
| North Africa, 2^nd^ generation | **1.42 (1.30-1.54)** | **1.16 (1.05-1.27)** | **1.11 (1.01-1.21)** | 1.05 (0.96-1.15) | **1.41 (1.29-1.54)** | **1.13 (1.02-1.25)** | 1.08 (0.98-1.20) | 1.03 (0.93-1.14) |
| Sub-Saharan Africa, 2^nd^ generation | **1.89 (1.35-2.65)** | **1.65 (1.21-2.25)** | **1.55 (1.13-2.12)** | **1.44 (1.05-1.98)** | **2.12 (1.42-3.16)** | 1.16 (0.80-1.70) | 1.10 (0.75-1.62) | 1.04 (0.71-1.54) |
| Asia, 2^nd^ generation | **1.31 (1.07-1.61)** | 1.17 (0.93-1.47) | 1.11 (0.88-1.40) | 1.06 (0.84-1.33) | **1.37 (1.09-1.71)** | 0.98 (0.76-1.27) | 0.94 (0.72-1.22) | 0.90 (0.69-1.16) |
| Other, 2^nd^ generation | **1.68 (1.39-2.03)** | **1.32 (1.08-1.61)** | **1.25 (1.03-1.52)** | 1.20 (0.98-1.47) | **1.83 (1.47-2.28)** | 1.09 (0.87-1.36) | 1.03 (0.82-1.29) | 1.00 (0.80-1.26) |
| **Stratification by Sex** |  |  |  |  |  |  |  |  |
| Female |  |  |  |  |  |  |  |  |
| Overseas territories, 1^st^ generation | **2.38 (1.81-3.14)** | **1.52 (1.21-1.92)** | **1.38 (1.09-1.75)** | **1.29 (1.01-1.64)** | **2.39 (1.74-3.28)** | 1.25 (0.97-1.62) | 1.13 (0.87-1.48) | 1.07 (0.82-1.40) |
| Europe, 1^st^ generation | **1.42 (1.26-1.59)** | 1.06 (0.94-1.19) | 1.02 (0.90-1.15) | 0.97 (0.86-1.10) | **1.44 (1.26-1.64)** | 0.98 (0.85-1.12) | 0.95 (0.82-1.09) | 0.90 (0.79-1.04) |
| North Africa, 1^st^ generation | **1.45 (1.13-1.87)** | **2.04 (1.61-2.57)** | **1.96 (1.55-2.49)** | **1.94 (1.53-2.46)** | **1.37 (1.03-1.81)** | **1.71 (1.31-2.23)** | **1.63 (1.25-2.14)** | **1.62 (1.23-2.13)** |
| Sub-Saharan Africa, 1^st^ generation | **1.58 (1.13-2.20)** | **1.59 (1.16-2.17)** | **1.61 (1.17-2.21)** | **1.53 (1.12-2.10)** | 1.42 (0.97-2.06) | 1.23 (0.86-1.77) | 1.28 (0.89-1.84) | 1.24 (0.86-1.79) |
| Asia, 1^st^ generation | 0.95 (0.74-1.22) | 1.12 (0.84-1.48) | 1.13 (0.86-1.50) | 1.15 (0.87-1.53) | 0.97 (0.73-1.30) | 0.92 (0.66-1.27) | 0.93 (0.67-1.29) | 0.95 (0.69-1.33) |
| Other, 1^st^ generation | **1.76 (1.41-2.20)** | 1.13 (0.91-1.41) | 1.06 (0.85-1.32) | 0.99 (0.79-1.24) | **1.66 (1.29-2.14)** | 1.07 (0.83-1.37) | 1.02 (0.79-1.31) | 0.96 (0.75-1.24) |
| Overseas territories, 2^nd^ generation | **2.14 (1.57-2.91)** | **1.40 (1.08-1.82)** | 1.29 (0.99-1.68) | 1.19 (0.92-1.55) | **2.15 (1.54-3.01)** | 1.14 (0.86-1.51) | 1.05 (0.79-1.39) | 0.97 (0.73-1.29) |
| Europe, 2^nd^ generation | **1.33 (1.23-1.44)** | **1.12 (1.03-1.22)** | 1.08 (0.99-1.17) | 1.05 (0.96-1.14) | **1.35 (1.24-1.47)** | 1.09 (0.99-1.19) | 1.05 (0.96-1.15) | 1.02 (0.93-1.12) |
| North Africa, 2^nd^ generation | **1.41 (1.26-1.58)** | 1.10 (0.97-1.23) | 1.05 (0.93-1.18) | 1.00 (0.89-1.12) | **1.37 (1.21-1.55)** | 1.06 (0.93-1.21) | 1.02 (0.90-1.16) | 0.97 (0.85-1.10) |
| Sub-Saharan Africa, 2^nd^ generation | **2.37 (1.43-3.95)** | **1.55 (1.04-2.31)** | 1.45 (0.97-2.17) | 1.34 (0.90-2.01) | **2.18 (1.25-3.82)** | 1.11 (0.69-1.81) | 1.05 (0.64-1.71) | 0.99 (0.60-1.62) |
| Asia, 2^nd^ generation | 1.25 (0.96-1.63) | 1.22 (0.93-1.60) | 1.15 (0.87-1.51) | 1.10 (0.84-1.45) | 1.30 (0.97-1.74) | 1.09 (0.81-1.49) | 1.04 (0.76-1.41) | 1.00 (0.73-1.35) |
| Other, 2^nd^ generation | **1.65 (1.28-2.11)** | **1.33 (1.04-1.68)** | 1.26 (0.99-1.61) | 1.20 (0.94-1.53) | **1.76 (1.32-2.37)** | 1.17 (0.89-1.54) | 1.11 (0.85-1.46) | 1.06 (0.81-1.40) |
| Male |  |  |  |  |  |  |  |  |
| Overseas territories, 1^st^ generation | **1.80 (1.37-2.37)** | **1.84 (1.37-2.47)** | **1.70 (1.27-2.29)** | **1.60 (1.18-2.16)** | **1.63 (1.21-2.22)** | **1.41 (1.00-1.98)** | 1.33 (0.94-1.87) | 1.26 (0.89-1.78) |
| Europe, 1^st^ generation | **1.24 (1.10-1.40)** | **1.19 (1.01-1.40)** | 1.15 (0.98-1.36) | 1.10 (0.93-1.30) | **1.22 (1.07-1.40)** | 1.06 (0.88-1.27) | 1.03 (0.85-1.24) | 0.99 (0.82-1.19) |
| North Africa, 1^st^ generation | **1.25 (1.04-1.52)** | **2.04 (1.64-2.54)** | **2.01 (1.61-2.50)** | **1.97 (1.58-2.46)** | 1.19 (0.96-1.47) | **1.60 (1.25-2.05)** | **1.59 (1.24-2.03)** | **1.57 (1.22-2.02)** |
| Sub-Saharan Africa, 1^st^ generation | **1.72 (1.31-2.24)** | **1.72 (1.28-2.31)** | **1.48 (1.08-2.02)** | **1.39 (1.02-1.91)** | **1.56 (1.16-2.11)** | 1.12 (0.80-1.58) | 0.97 (0.67-1.40) | 0.93 (0.64-1.34) |
| Asia, 1^st^ generation | **1.38 (1.01-1.90)** | **1.88 (1.32-2.66)** | **1.75 (1.23-2.49)** | **1.72 (1.20-2.46)** | 1.22 (0.87-1.72) | **1.88 (1.30-2.73)** | **1.75 (1.20-2.56)** | **1.74 (1.20-2.53)** |
| Other, 1^st^ generation | **1.99 (1.50-2.63)** | **1.38 (1.01-1.89)** | 1.24 (0.90-1.71) | 1.12 (0.80-1.55) | **2.01 (1.47-2.74)** | 1.21 (0.85-1.73) | 1.10 (0.77-1.58) | 1.01 (0.70-1.45) |
| Overseas territories, 2^nd^ generation | **1.53 (1.12-2.10)** | 1.38 (0.96-1.99) | 1.24 (0.85-1.82) | 1.15 (0.78-1.69) | **1.56 (1.11-2.19)** | 1.15 (0.76-1.74) | 1.04 (0.68-1.61) | 0.97 (0.63-1.51) |
| Europe, 2^nd^ generation | **1.15 (1.06-1.24)** | **1.16 (1.04-1.29)** | **1.15 (1.04-1.28)** | **1.13 (1.02-1.26)** | **1.14 (1.05-1.24)** | 1.10 (0.98-1.23) | 1.10 (0.98-1.23) | 1.09 (0.97-1.22) |
| North Africa, 2^nd^ generation | **1.43 (1.27-1.61)** | **1.26 (1.08-1.46)** | **1.19 (1.03-1.39)** | 1.14 (0.98-1.33) | **1.45 (1.27-1.65)** | **1.24 (1.06-1.46)** | **1.19 (1.02-1.40)** | 1.15 (0.98-1.35) |
| Sub-Saharan Africa, 2^nd^ generation | 1.52 (0.95-2.41) | **1.83 (1.13-2.94)** | **1.73 (1.07-2.82)** | **1.64 (1.00-2.67)** | **2.03 (1.14-3.61)** | 1.28 (0.70-2.34) | 1.24 (0.68-2.26) | 1.17 (0.64-2.15) |
| Asia, 2^nd^ generation | **1.41 (1.02-1.96)** | 1.06 (0.69-1.63) | 1.03 (0.67-1.57) | 0.96 (0.63-1.47) | **1.46 (1.02-2.09)** | 0.77 (0.46-1.29) | 0.75 (0.45-1.26) | 0.71 (0.42-1.18) |
| Other, 2^nd^ generation | **1.73 (1.29-2.32)** | 1.30 (0.92-1.84) | 1.21 (0.85-1.73) | 1.20 (0.84-1.71) | **1.91 (1.37-2.68)** | 0.93 (0.62-1.40) | 0.87 (0.58-1.32) | 0.87 (0.58-1.32) |
